# Increased lactate dehydrogenase reflects the progression of COVID-19 pneumonia on chest computed tomography and predicts subsequent severe disease

**DOI:** 10.1038/s41598-023-28201-2

**Published:** 2023-01-18

**Authors:** Kensuke Kojima, Hyungeun Yoon, Kyoichi Okishio, Kazunari Tsuyuguchi

**Affiliations:** 1 Department of General Thoracic Surgery, National Hospital Organization Kinki-Chuo Chest Medical Center, 1180 Nagasone-cho, Kita-ku, Sakai, Osaka 591-8555 Japan; 2 Department of Internal Medicine, National Hospital Organization Kinki-Chuo Chest Medical Center, Osaka, Japan; 3 Department of Infectious Diseases, National Hospital Organization Kinki-Chuo Chest Medical Center, Osaka, Japan; 4 Clinical Research Center, National Hospital Organization Kinki-Chuo Chest Medical Center, Osaka, Japan

**Keywords:** Medical research, Risk factors

## Abstract

Chest computed tomography (CT) is effective for assessing the severity of coronavirus disease 2019 (COVID-19). However, the clinical factors reflecting the disease progression of COVID-19 pneumonia on chest CT and predicting a subsequent exacerbation remain controversial. We conducted a retrospective cohort study of 450 COVID-19 patients. We used an automated image processing tool to quantify the COVID-19 pneumonia lesion extent on chest CT at admission. The factors associated with the lesion extent were estimated by a multiple regression analysis. After adjusting for background factors by propensity score matching, we conducted a multivariate Cox proportional hazards analysis to identify factors associated with severe disease after admission. The multiple regression analysis identified, body-mass index (BMI), lactate dehydrogenase (LDH), C-reactive protein (CRP), and albumin as continuous variables associated with the lesion extent on chest CT. The standardized partial regression coefficients for them were 1.76, 2.42, 1.54, and 0.71. The multivariate Cox proportional hazards analysis identified LDH (hazard ratio, 1.003; 95% confidence interval, 1.001–1.005) as a factor independently associated with the development of severe COVID-19 pneumonia. Increased serum LDH at admission may be useful in real-world clinical practice for the simple screening of COVID-19 patients at high risk of developing subsequent severe disease.

## Introduction

The outbreak of coronavirus disease 2019 (COVID-19) due to severe acute respiratory syndrome coronavirus 2 (SARS-CoV-2) at the end of 2019 rapidly spread worldwide and caused a pandemic. Although novel messenger RNA (mRNA) vaccines against COVID-19 have been developed and vaccination is underway worldwide, the pandemic has not yet ended due to the emergence of mutant strains.

Most patients infected with COVID-19 have a relatively mild clinical course, but some patients develop serious symptoms, including acute respiratory distress syndrome (ARDS) and death^1^. Therefore, understanding which patients are more likely to develop severe disease due to COVID-19 infection is an important clinical issue. Some factors, such as comorbidity and laboratory data, have been reported to be associated with severity of COVID-19 infection^2–5^. In addition, several candidate risk factors have been also reported in analyses of large cohort from previous studies^6,7^. In COVID-19, chest computed tomography (CT) has been reported to be useful for stratifying the severity of lung lesions and predicting the prognosis^8^. The CT severity score—semiquantitative scoring of lung parenchyma lesions by radiologist—estimated based on chest CT finding has also been reported to be useful as a predictor of severity of COVID-19 pneumonia, and some studies have reported an association between the CT severity score and inflammatory markers^9^. However, consistent information is lacking regarding the clinical factors that predict the COVID-19 lesion extent within total lung on chest CT and how those factors are associated with severe COVID-19 pneumonia during the clinical course.

In the present study, we used an image analysis system to automatically quantify the extent of inflammatory images concerning COVID-19 pneumonia on chest CT and estimated the factors associated with that numerical values by a multiple regression analysis. In addition, we investigated the relationship between those factors and the development of severe COVID-19 pneumonia by a multivariate Cox hazard analysis. This knowledge may be used to assess and predict the severity of COVID-19 pneumonia with simple information such as blood test values and comorbidities. In addition, it may aid in appropriately screening patients who need treatment and determining the immediate provision of medical care in real-world clinical practice, especially in the current situation where the healthcare system is under pressure due to the COVID-19 pandemic.

## Results

### Patient characteristics

The patient characteristics are shown in Table 1. Of the 450 patients, 66.4% were men. The median age, BMI, and the days from the onset to admission were 58 years old, 24.2 kg/m^2^ and 6.0 days, respectively. Regarding comorbidities, 18.0% of patients had diabetes mellitus, 34.4% hypertension, 6.6% chronic obstructive pulmonary disease, 18.2% hyperlipidemia, 10.0% coronary heart disease, 5.1% cerebrovascular disease, 3.8% chronic kidney disease, and 8.7% cancer. The median values of laboratory test findings at admission for white blood cells, lymphocytes, neutrophils, LDH, CRP, ferritin, D-dimer, albumin, AST, ALT, and creatinine were 5.2 × 10^3^/μL, 0.9 × 10^3^/μL, 3.7 × 10^3^/μL, 304 IU/L, 5.4 mg/dL, 532 ng/mL, 1.2 μg/mL, 3.7 g/dL, 38 IU/L, 30 IU/L, and 0.9 mg/dL, respectively. The percentages of GGO and consolidation to total lung volume on chest CT at admission were 22.9% and 1.1%, respectively. The administered medications were dexamethasone, systemic corticosteroid, remdesivir, tocilizumab, and baricitinib, with usage rates of 54%, 25%, 72%, 9%, and 12%, respectively.

**Table 1:** Baseline characteristics of patients with COVID-19.

Characteristics	Overall (n = 450)	Non-severe (n = 385)	Severe (n = 65)	*P*
Demographics				
Age—median (range, Q1–Q3) (years)	58 (47–73)	56 (46–72)	68 (58–74)	< 0.001*
Male sex—no. (%)	299 (66.4)	251 (65.2)	48 (73.8)	0.22^†^
Current/former smoker—no. (%)	230 (51.1)	192 (49.9)	38 (58.5)	0.25^†^
BMI—median (range, Q1–Q3) (kg/m^2^)	24.2 (21.8–27.4)	23.9 (21.7–27.3)	25.1 (22.5–27.5)	0.09^‡^
Days from the onset to admission—median (range, Q1–Q3)	6.0 (4.0–8.0)	6.0 (4.0–8.0)	7.0 (4.0–8.0)	0.12^‡^
Comorbidity—no. (%)				
Diabetes mellitus	81 (18.0)	62 (16.1)	19 (29.2)	0.02^†^
Hypertension	155 (34.4)	128 (33.2)	27 (41.5)	0.25^†^
COPD	30 (6.6)	23 (6.0)	7 (10.8)	0.18^§^
Hyperlipidemia	82 (18.2)	70 (18.2)	12 (18.5)	1.00^†^
Coronary heart disease	45 (10.0)	34 (8.8)	11 (16.9)	0.07^†^
Cerebrovascular disease	23 (5.1)	22 (5.7)	1 (1.5)	0.23^§^
Chronic kidney disease	17 (3.8)	13 (3.4)	4 (6.2)	0.23^§^
Cancer	39 (8.7)	26 (6.8)	13 (20.0)	0.001^§^
Laboratory data at admission—median (range, Q1–Q3)				
White blood cells (× 10^3^/μL)	5.2 (4.1–6.7)	5.1 (4.1–6.6)	5.6 (4.3–7.4)	0.22^‡^
Lymphocytes (× 10^3^/μL)	0.9 (0.6–1.2)	0.9 (0.7–1.2)	0.7 (0.6–1.0)	< 0.001^‡^
Neutrophils (× 10^3^/μL)	3.7 (2.6–5.1)	3.5 (2.6–5.1)	4.5 (3.0–6.3)	0.02^‡^
LDH (IU/L)	304 (235–405)	285 (226–377)	453 (360–531)	< 0.001^‡^
CRP (mg/dL)	5.4 (1.8–9.3)	4.4 (1.3–8.4)	9.9 (5.9–13.4)	< 0.001^‡^
Ferritin (ng/mL)	532 (242–1008)	472 (222–882)	1000 (687–1758)	< 0.001^‡^
D-dimer (μg/mL)	1.2 (0.9–1.6)	1.1 (0.8–1.5)	1.5 (1.3–2.0)	< 0.001^‡^
Albumin (g/dL)	3.7 (3.3–4.1)	3.8 (3.4–4.1)	3.5 (3.2–3.7)	< 0.001^‡^
AST (IU/L)	38 (27–58)	37 (26–51)	60 (44–72)	< 0.001^‡^
ALT (IU/L)	30 (18–55)	29 (17–53)	39 (22–57)	0.03^‡^
Creatinine (mg/dL)	0.9 (0.7–1.1)	0.9 (0.7–1.1)	1.0 (0.8–1.2)	0.002^‡^
Chest CT features at admission—median (range, Q1–Q3) (%)				
Ground-glass opacity	22.9 (14.9–32.1)	20.8 (13.8–30.3)	33.8 (26.3–40.8)	< 0.001^‡^
Consolidation	1.1 (0.8–2.4)	1.0 (0.7–2.2)	1.6 (1.0–4.5)	< 0.001^‡^
Treatments during hospitalization				
Dexamethasone	206 (54)	174 (45)	32 (49)	0.59^†^
Systemic corticosteroid	111 (25)	68 (18)	43 (66)	< 0.001^†^
Remdesivir	324 (72)	269 (70)	55 (85)	0.016^†^
Tocilizumab	42 (9)	22 (6)	20 (31)	< 0.001^†^
Baricitinib	56 (12)	45 (12)	11 (17)	0.23^†^
Oxygen administration	169 (38)	113 (29)	56 (88)	< 0.001^†^

*Welch’s *t* test, ^†^Pearson’s Chi-square test, ^‡^Mann–Whitney *U* test, ^§^Fisher’s exact test.

*ALT* alanine aminotransferase, *AST* aspartate aminotransferase, *BMI* body mass index, *COPD* chronic obstructive pulmonary disease, *COVID-19* coronavirus disease 2019, *CRP* C-reactive protein, *LDH* lactate dehydrogenase.

### Factors associated with GGO and consolidation on chest CT of COVID-19 pneumonia patients

We explored factors associated with the proportion of GGO and consolidation to total lung by a multiple regression analysis (Table 2). There was no multicollinearity in any of the explanatory valuables in the multiple regression analysis (VIF < 10). The normality of the residuals was assessed with a Residuals vs. Fitted and a Normal Q–Q plot (Supplementary Fig. 1). We confirmed that most of the data generally showed vertical symmetry (Supplementary Fig. 1a) and a 45° line (Supplementary Fig. 1b). Significant differences were noted for the following factors: female sex (partial regression coefficient: B, 1.11; 95% confidence interval [CI], 1.02–1.21), the BMI (B, 4.19; 95% CI 2.65–6.66), LDH (B, 3.38; 95% CI 2.33–4.89), CRP (B, 1.18; 95% CI 1.08–1.29), and albumin (B, 0.36; 95% CI 0.21–0.63). The strength of the association of continuous variable (i.e. BMI, LDH, CRP, and albumin) with GGO and consolidation of COVID-19 pneumonia was compared using the standardized partial regression coefficient (β), showing values of 1.76, 2.42, 1.54, and 0.71, respectively.

**Table 2:** Association between progression of COVID-19 pneumonia and baseline characteristics according to multiple regression analyses.

	B (95% CI)	β	*P*	VIF
Categorical variables				
Female sex	1.11 (1.02–1.21)	–	0.01	1.32
Diabetes mellitus	1.04 (0.94–1.14)	–	0.46	1.13
Hypertension	0.96 (0.88–1.05)	–	0.45	1.35
Continuous variables				
Age	0.97 (0.71–1.35)	0.99	0.90	1.76
BMI (kg/m^2^)	4.19 (2.65–6.66)	1.76	< 0.001	1.38
LDH (IU/L)	3.38 (2.33–4.89)	2.42	< 0.001	2.99
CRP (mg/dL)	1.18 (1.08–1.29)	1.54	< 0.001	2.36
Ferritin (ng/mL)	1.04 (0.93–1.17)	0.91	0.45	2.51
D-dimer (μg/mL)	0.96 (0.78–1.16)	0.96	0.65	1.63
Albumin (g/dL)	0.36 (0.21–0.63)	0.71	< 0.001	1.46
AST (U/L)	0.93 (0.68–1.28)	0.93	0.66	4.87
ALT (U/L)	0.93 (0.74–1.17)	0.91	0.56	4.46
White blood cells (/μL)	0.97 (0.58–1.62)	0.98	0.92	6.69
Lymphocytes (/μL)	0.90 (0.72–1.13)	0.91	0.36	1.69
Neutrophils (/μL)	1.17 (0.81–1.70)	1.18	0.40	6.32
Days from the onset to admission	1.08 (0.94–1.24)	1.11	0.26	1.41

Adjusted R^2^ 0.47, *P*-value < 0.001.

Objective variable and continuous variables in the explanatory variables were log-transformed to approximate a normal distribution for the multiple regression analysis. Therefore, the partial regression coefficient (B) and standardized partial regression coefficient (β) are shown to the power of 10.

*ALT* alanine aminotransferase, *AST* aspartate aminotransferase, *B* partial regression coefficient, *β* standardized partial regression coefficient, *BMI* body mass index, *COPD* chronic obstructive pulmonary disease, *COVID-19* coronavirus disease 2019, *CRP* c-reactive protein, *LDH* lactate dehydrogenase, *VIF* variance inflation factor.

### Adjustment of background factors

To examine the association of the five factors that showed significant differences in a multiple regression analysis with the subsequent development of severe COVID19 pneumonia, we adjusted for background factors by propensity score matching. Age, comorbidities (i.e. diabetes mellitus and hypertension), laboratory data at admission (i.e. white blood cells, lymphocytes, neutrophils, ferritin, D-dimer, AST, ALT, and creatinine), and treatments during hospitalization (i.e. dexamethasone, systemic corticosteroid, remdesivir, tocilizumab, and baricitinib) were included as covariates in the logistic regression model with the outcome of severe COVID-19 to calculate the propensity score. The results of propensity score matching are shown in Table 3. The variable balances after propensity score matching were all < 0.1, as evaluated by the standardized difference. After propensity score matching, the significant difference in covariates between the non-severe and severe groups was eliminated.

**Table 3:** Characteristics of matched and unmatched patients according to propensity score matching.

	Before matching			After matching			
	Non-severe (n = 385)	Severe (n = 65)	*P*	Non-severe (n = 51)	Severe (n = 51)	*P*	Std diff
Demographics							
Age (years)	56 (46–72)	68 (58–74)	< 0.001*	66 (57–72)	68 (58–73)	0.63^||^	0.09
Days from the onset to admission	6 (4–8)	7 (4–8)	0.12^‡^	7 (5–8)	8 (4–8)	0.73^‡^	0.01
Laboratory data at admission							
White blood cells (× 10^3^/μL)	5.1 (4.1–6.6)	5.6 (4.3–7.4)	0.22^‡^	5.2 (4.2–6.9)	5.4 (4.2–7.2)	0.78^‡^	0.08
Lymphocytes (× 10^3^/μL)	0.9 (0.7–1.2)	0.7 (0.6–1.0)	< 0.001^‡^	0.7 (0.5–0.9)	0.7 (0.6–0.9)	0.45^‡^	0.05
Neutrophils (× 10^3^/μL)	3.5 (2.6–5.1)	4.5 (3.0–6.3)	0.02^‡^	3.9 (2.9–5.6)	3.9 (2.9–5.3)	0.88^‡^	0.09
Ferritin (× 10^3^ ng/mL)	0.5 (0.2–0.8)	1.0 (0.7–1.7)	< 0.001^‡^	0.9 (0.7–1.9)	0.9 (0.6–1.7)	0.53^‡^	0.08
D-dimer (μg/mL)	1.1 (0.8–1.5)	1.5 (1.3–2.0)	< 0.001^‡^	1.5 (1.1–2.0)	1.4 (1.3–1.9)	0.75^‡^	0.06
AST (IU/L)	37 (26–51)	60 (44–72)	< 0.001^‡^	45 (38–78)	60 (45–72)	0.20^‡^	0.01
ALT (IU/L)	29 (17–53)	39 (22–57)	0.03^‡^	38 (23–67)	37 (22–57)	0.78^‡^	0.02
Creatinine (mg/dL)	0.9 (0.7–1.1)	1.0 (0.8–1.2)	0.002^‡^	0.9 (0.8–1.2)	1.0 (0.8–1.1)	0.83^‡^	0.10
Comorbidity							
Diabetes mellitus	62 (16)	19 (29)	0.02^†^	14 (28)	15 (29)	1.00^§^	0.04
Hypertension	128 (33)	27 (41)	0.25^†^	21 (41)	23 (45)	0.84^†^	0.07
Treatments during hospitalization							
Dexamethasone	174 (45)	32 (49)	0.59^†^	29 (57)	28 (55)	1.00^†^	0.04
Systemic corticosteroid	68 (18)	43 (66)	< 0.001^†^	31 (61)	32 (63)	1.00^†^	0.04
Remdesivir	269 (70)	55 (85)	0.016^†^	48 (94)	47 (92)	1.00^†^	0.07
Tocilizumab	22 (6)	20 (31)	< 0.001^†^	10 (20)	12 (23)	0.81^§^	0.09
Baricitinib	45 (12)	11 (17)	0.23^†^	11 (22)	11 (22)	1.00^§^	0.001

Data are presented as the median (IQR) or n (%).

*Welch’s *t* test, ^†^Pearson’s Chi-square test, ^‡^Mann–Whitney *U* test, ^§^Fisher’s exact test, ^||^Student’s *t* test.

*ALT* alanine aminotransferase, *AST* aspartate aminotransferase, *Std* standardized difference.

### Factors associated with the development of severe COVID-19 pneumonia

A Cox proportional hazards analysis was used as a multivariate analysis of the survival in the matched sample (i.e. 51 patients in the non-severe group and 51 patients in the severe group). We set the outcome as the number of days from symptom awareness to severe disease, and the explanatory variables were LDH (continuous variable), CRP (continuous variable), albumin (continuous variable), BMI (continuous variable), and sex (categorical variable).

**Figure 1:**
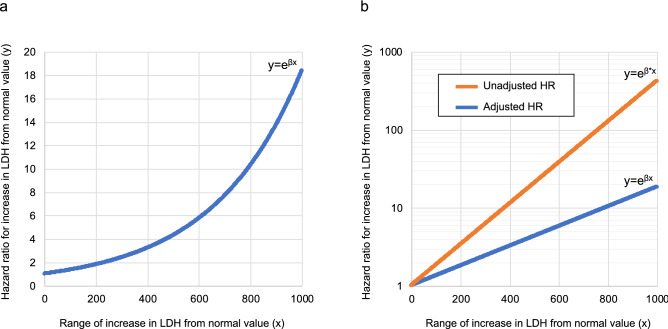
(**a**,**b**) Hazard ratios of COVID-19 pneumonia severity for each increase in LDH level from the normal value. (**a**) The hazard ratio (y) for each LDH level relative to the range of increase from the normal value (x) was calculated based on the results of a multivariate Cox proportional hazards analysis. Since the regression coefficient (β) for this multivariate analysis was calculated to be 0.00299551, the hazard ratio was given by the value of e^β^, i.e. 1.003. The function y was given by y = e^βx^. (**b**) A comparison of the adjusted and unadjusted hazard ratios of a development of severe COVID-19 pneumonia for each range of increase in serum LDH levels from the normal value. The y-axis has been transformed to a logarithmic scale. e^β^ and e^β*^ are given as 1.003 and 1.006, respectively. *β* regression coefficient for the multivariate analysis, β^*^ regression coefficient for the univariate analysis.

The results of the Cox proportional hazards analysis of factors associated with severe disease are shown in Table 4. Significant differences were observed in LDH (adjusted hazard ratio [HR], 1.003; 95% CI 1.001–1.005). However, while CRP showed a significant difference in the unadjusted HR (1.07; 95% CI 1.05–1.10), the difference was no longer significant in the adjusted HR (0.99; 95% CI 0.94–1.02). Based on the HR of LDH, the results of plotting the HR increase from the normal value of LDH in the range of 0–1000 are shown in Fig. 1a. The graph showed that the HR increases from 1.003 to 1.8 for a 200 increase in the LDH level from the normal value. In addition, when the LDH level increases by 400, 600, 800, and 1000 from the normal value, the HRs increased from 3.2, 5.7, and 10.3 to 18.4, respectively. Based on the unadjusted HRs of LDH, the results of plotting each HR against the increase from the normal value of LDH are shown in Fig. 1b, along with a graph of the adjusted HRs. The values differed greatly between the adjusted and unadjusted HRs.

**Table 4:** Risk factors for the development of severe COVID-19 pneumonia.

	Unadjusted HR^a^ (95% CI), *P*	Adjusted HR^b^ (95% CI), *P*
Categorical variable		
Female sex	0.66 (0.38–1.15), 0.14	1.67 (0.87–3.20), 0.13
Continuous variable		
LDH (IU/L)	1.006 (1.005–1.007), < 0.001	1.003 (1.001–1.005), 0.002
CRP (mg/dL)	1.07 (1.05–1.10), < 0.001	0.99 (0.94–1.02), 0.40
Albumin (g/dL)	0.51 (0.36–0.73), < 0.001	1.85 (0.94–3.65), 0.08
BMI (kg/m^2^)	1.03 (0.99–1.07), 0.17	1.04 (0.99–1.08), 0.13

^a^A univariate Cox proportional hazards analysis using 450 cases before adjusting for propensity score matching.

^b^A multivariate Cox proportional hazards analysis using 102 cases after adjusting for propensity score matching.

*BMI* body mass index, *COVID-19* coronavirus disease 2019, *CRP* c-reactive protein, *LDH* lactate dehydrogenase.

## Discussion

In the univariate analysis, age, BMI, lymphocytes, neutrophils, LDH, CRP, ferritin, D-dimer, albumin, AST, ALT, and creatinine were significantly associated with severe COVID-19. These results show a similar trend to previous studies^2,6^ and indicates that the cohort we used for our study is not unique. The percentage of GGO and consolidation within total lung on chest CT on COVID-19 pneumonia at admission was quantified using an imaging tool. A multiple regression analysis of the factors associated with that proportions showed that the associated factors were sex, BMI, LDH, CRP, and albumin. The association of the development of severe COVID-19 pneumonia with these five factors was analyzed by a multivariate Cox proportional hazards analysis using a sample adjusted for background factors by propensity score matching. According to the results, only LDH showed a significant difference, indicating that for every 1 increase in LDH from the normal value, the risk of severity increased 1.003-fold. In other words, the risk increased exponentially by 2-, 3-, 6-, 10-, and 18-fold as the LDH increased by 200, 400, 600, 800, and 1000 above the normal value.

The search for clinical factors associated with the severity, i.e. application of mechanical ventilation and mortality, of COVID-19 has been the subject of many previous studies^1–5^. However, many of those studies were based on univariate analyses and did not adequately adjust for background factors. Some studies using large cohorts, in which background factors were adequately adjusted in multivariate analyses, have been reported^6,7^. However, inflammatory markers, such as CRP and LDH, which have been frequently reported to be associated with the severity of COVID-19^10,11^, were not included in those analyses. In addition, there have been reported that chest CT is useful for assessing the severity of COVID-19^12,13^. However, few studies have evaluated the association between COVID-19 severity and chest CT by a multivariate analysis with adequate adjustment for background factors. Few studies have employed a method adequately adjusting for background factors to identify clinical factors associated with the COVID-19 pneumonia lesion extent within the total lung on chest CT at admission and predicted severe disease after admission.

There were two novel points associated with the present study. First, we identified the details of clinical factors associated with the progression of COVID-19 pneumonia on chest CT at admission using a multiple regression analysis. We automatically quantified the extent of COVID-19 pneumonia on chest CT using an image analysis system. The fact that we used that numerical values as the outcome of the multivariate analysis is very different from conventional studies using CT severity scores as the outcome, which rely on a subjective semiquantitative evaluation by radiologists^9^. Using our objective quantitative evaluation, we found that the five factors of sex, BMI, LDH, CRP, and albumin were significantly associated with progression of COVID-19 pneumonia on chest CT at admission.

Women reportedly have a higher incidence of ARDS than men^14^. However, a study on the association between COVID-19 pneumonia and CT severity score reported no significant difference in sex^15^. In our multiple regression analysis, the partial regression coefficient was as low as 1.11, so the difference in COVID-19 pneumonia severity based on sex on chest CT may not be a problem in real-world clinical practice.

The four other factors of BMI, LDH, CRP, and albumin, have also been reported to be associated with ARDS. Obesity is known to promote an inflammatory response and the endothelial changes seen in ARDS^16^. Since there are reports suggesting an association between COVID-19 pneumonia and the BMI^17^, obese patients may be prone to elevated inflammatory adipokines induced by COVID-19 infection. Inflammatory markers, such as LDH and CRP, have been reported to be useful in predicting the early onset of ARDS and its prognosis^18,19^. Similarly, many studies have suggested that LDH and CRP may be prognostic factors for severity of COVID-19^20,21^. Hypoalbuminemia has been suggested to be associated with ARDS, as it causes increased alveolar capillary permeability and promotes edema formation^22^. Our multiple regression analysis showed a significant inverse correlation between albumin levels and COVID-19 pneumonia, suggesting that edema related to vascular permeability associated with hypoalbuminemia may contribute to the lesion extent of COVID-19 pneumonia on chest CT.

A comparison of the standardized partial regression coefficients of these four factors obtained by a multiple regression analysis revealed that LDH, BMI, CRP, and albumin, in that order, were strongly associated with disease progression of COVID-19 pneumonia. Although these factors have been analyzed and compared as categorical variables in previous studies, we treated them as continuous variables and directly compared the strength of the association. As a result, we revealed for the first time that LDH is the most suitable factor for assessing the COVID-19 pneumonia lesion extent within total lung on chest CT at admission. Since LDH is an enzyme contained in cells that catalyzes the conversion of pyruvate to lactate—the final step of anaerobic glycolysis—and elevated LDH indicates the degree of cell damage associated with tissue hypoperfusion, LDH may most directly reflect the extent of lung damage.

The second novel point of our study is that we found that LDH is a potential predictor of severe disease after admission. In our univariate analysis without considering the effect of confounders, the factors LDH, CRP, and albumin were significantly associated with the development of severe COVID-19 pneumonia. In contrast, our multivariate analysis using a cohort adjusted for background factors, including those factors suggested to be associated with COVID-19 severity in a previous study^6^, by propensity score matching showed that only LDH was significantly associated with the subsequent development of severe COVID-19 pneumonia in the clinical course. Although CRP, albumin, and BMI can be used to assess the disease progression of COVID-19 pneumonia on chest CT at admission, they may not be predictors of severe disease after admission.

Many previous studies have reported the association between the severity of COVID-19 including mortality and LDH^23^. However, LDH was treated as a categorical variable, and the cut-off value was set independently by each study, so the results varied among studies. Therefore, it would be difficult to use the results as a specific indicator of risk of COVID-19 severity in real-world clinical practice. We overcame this problem by treating LDH as a continuous variable. We showed that the hazard ratio of LDH for a development of severe COVID-19 pneumonia was 1.006 in a univariate analysis and 1.003 in a multivariate analysis using a propensity score-matched cohort. This is a small difference, but it indicated that the larger the increase in LDH from the normal value, the larger this difference becomes, indicating that overestimation was controlled by adjustment for background factors. No study has ever evaluated LDH as a continuous variable and specifically reported the risk of COVID-19 severity according to individual LDH data points. It may be possible to predict the risk of a development of severe COVID-19 pneumonia after admission simply by measuring the LDH value at admission.

We showed that LDH represents the COVID-19 pneumonia lesion extent within total lung at admission and may be a predictor of severe disease after admission. This mechanism may have a biological explanation. Although the details of COVID-19 infection and cytotoxicity are not known and are the subject of active research, recent experiments using lung organoids have reported that cells die after infection with COVID-19 and that the virus may induce cytokine storm and cause cytotoxicity^24,25^. In other words, the cell death caused by COVID-19 infection of lung cells may lead to extracellular release of LDH and induce a direct increase in serum LDH. However, studies on the route of entry of COVID-19 have suggested that COVID-19 may enter the lungs via the respiratory tract and then spread throughout the body via vascular endothelial cells, entering other organs^26^. In other words, the virus spreads from the lungs to systemic organs and induces a cytokine storm, which is thought to cause multi-organ damage. Through this indirect cellular damage, extracellular release of LDH is also triggered, which is expected to increase the level. CRP is a protein produced in the liver^27^ and adipocytes^28^ in response to inflammatory cytokines such as IL-6, and is thought to reflect the intensity of inflammation or the degree of cytokine storm. It may be an indicator of indirect cytotoxicity like LDH, but it does not reflect the degree of direct cytotoxicity like LDH, so it may be less effective than LDH for evaluating the degree and prognosis of COVID-19 pneumonia. Similarly, the BMI and albumin level are indirectly related to inflammation^29,30^, but they do not reflect direct cellular damage, so they may be less effective as assessment factors than LDH.

Several limitations associated with the present study warrant mention. First, this was a single-center study, which limits the sample size. Since a multivariate analysis is dependent on the sample size and the sample size of the adjusted cohort will be reduced by propensity score matching, further validation in a larger cohort may be necessary. Second, all cases used in this study were patients with mild or moderate symptoms at admission, and patients with severe symptoms were not included. It is therefore necessary to evaluate whether or not similar results can be obtained in patients with severe symptoms at admission. Third, no distinction was made between the mutant strains of COVID-19. Judging from the timing of our study and the prevalent variants of COVID-19 in Japan, it is likely that majority of patients were infected with B.1.1.7 (Alpha strain according to the World Health Organization classification)^31^ or B.1.617.2 (Delta strain)^32^. There have been reports of differences in viral load depending on the strain and consequent differences in severity depending on the viral load^33^; it may therefore be necessary to include the type of mutant strains as a variable in multivariate analyses. Fourth, the pneumonia imaging findings in the automated image analysis of chest CT may have been overanalyzed as COVID-19 pneumonia, since bacterial and viral pneumonia cannot be distinguished in this manner. Although the settings for the automatic image analysis were based on those used in several previous studies, the accumulation of COVID-19 pneumonia imaging may require more COVID-19-specific image analysis settings. However, in our multivariate analysis, we included factors that are commonly elevated in bacterial pneumonia, i.e. white blood cell count, neutrophil count, and CRP, so we believe that we were able to reduce this effect statistically.

In summary, our results suggested that an increased serum LDH level at admission was an independent risk factor that reflected the extent of lesion induced by COVID-19 pneumonia on chest CT and predicted severe disease after admission in patients with mild to moderate COVID-19. The serum LDH level at admission in COVID-19 patients with mild to moderate disease may aid in the early identification of COVID-19 pneumonia patients likely to develop severe disease in the subsequent clinical course in a non-invasive and simple manner without the need for chest CT.

## Methods

### Patients

This study was a retrospective, single-center cohort study of patients with laboratory-confirmed COVID-19 infection between October 1, 2020, and September 23, 2021. Our hospital admitted patients who were judged to have mainly mild to moderate disease by follow-up centers or health centers contacted by patients with COVID-19 infection recuperating at home, in hotel rooms, or in geriatric health facilities. The definitive diagnosis of COVID-19 infection was based on the results of reverse transcription polymerase chain reaction (RT-PCR) or antigen testing at the Osaka Regional Health Center. All patients underwent CT of the chest at admission, and their height and weight were measured. Patients whose height and weight data were not available were excluded. In addition, patients who had received even one dose of an mRNA vaccine against COVID-19 were also excluded. The medical records of a total of 450 patients with COVID-19 were analyzed retrospectively to determine the clinical factors associated with the severity of COVID-19 pneumonia. Clinical and laboratory data, such as the age, sex, comorbidities, number of days from the onset to admission, height, weight, blood tests, chest CT findings, treatment after admission, and outcome, were collected from all patients. The date of the onset was defined as the date when the patient became clearly aware of clinical symptoms, such as a fever, cough, and fatigue, determined from a medical interview; the days from the onset to admission were then estimated. All patients were admitted to the COVID-19-infected unit in our hospital. In addition to basic supportive care, patients admitted to the unit received treatment with medication, oxygenation, and mechanical ventilation, as indicated. More specifically, patients were treated according to their percutaneous oxygen saturation (SpO_2_) on admission. Patients with mild COVID-19 (SpO_2_ > 96%) were treated with symptomatic therapy. Regarding COVID-19-specific drugs, patients with moderate I disease (93% < SpO_2_ < 96%) were treated with baricitinib, dexamethasone, and remdesivir, while patients with moderate I to II disease (SpO_2_ < 93%) were treated with dexamethasone, remdesivir, systemic corticosteroid therapy, and tocilizumab, as appropriate. Oxygen was administered if the percutaneous oxygen saturation on room air breathing was continuously below 93%. High-flow oxygen therapy was applied if the increase in oxygen saturation was poor. If the oxygen saturation could not be maintained above 90% even with high-flow oxygen administration, it was determined that mechanical ventilation was applicable. Severe COVID-19 in our study was defined as a condition in which mechanical ventilation was judged to be necessary after hospitalization. Patients who were indicated to be managed by a mechanical ventilator were transferred to a hospital specializing in critical care. Patients who were judged to require management by a mechanical ventilator were classified into the severe group, including those who were transferred out. All other patients were classified into the non-severe group. Our study was approved by the Institutional Review Board of Kinki-Chuo Chest Medical Center (Approval Number: 2022-039). Informed consent was obtained by an opt-out method using the website of our institution. All methods were performed in accordance with relevant guidelines and regulations.

### Image analyses

All patients with COVID-19 infection underwent chest CT scan at admission. On CT, COVID-19 pneumonia is characterized by ground-glass opacity (GGO) extending into the lung fields along with areas of consolidation. Quantification of the areas of the lung affected by COVID-19 pneumonia was performed using an image processing support tool (Synapse VINCENT version 5; Fujifilm Corporation, Tokyo, Japan). Synapse VINCENT allows for the separation of the lung from the rest of the chest and provides an analysis of the distribution of pixels on CT expressed as a percentage of the total lung volume according to their density. This imaging tool is usually used to assess the emphysema proportion (low-density pixels), but can be used for other density ranges as well. The density range to quantify the high-density pixels typical of COVID-19 lung lesions on CT was manually set in SYNAPSE VINCENT by referencing to previous studies^34,35^, creating the COVID-19 dataset, which consists of 4 groups based on the following Hounsfield unit (HU) classifications: values from − 1024 HU to − 950 HU (red) representing emphysema; values from − 949 HU to − 750 HU (yellow) representing healthy lung tissue; − 749 HU to − 300 HU (blue) representing GGO; and values from − 299 HU to 40 HU (violet) representing consolidation.

**Figure 2:**
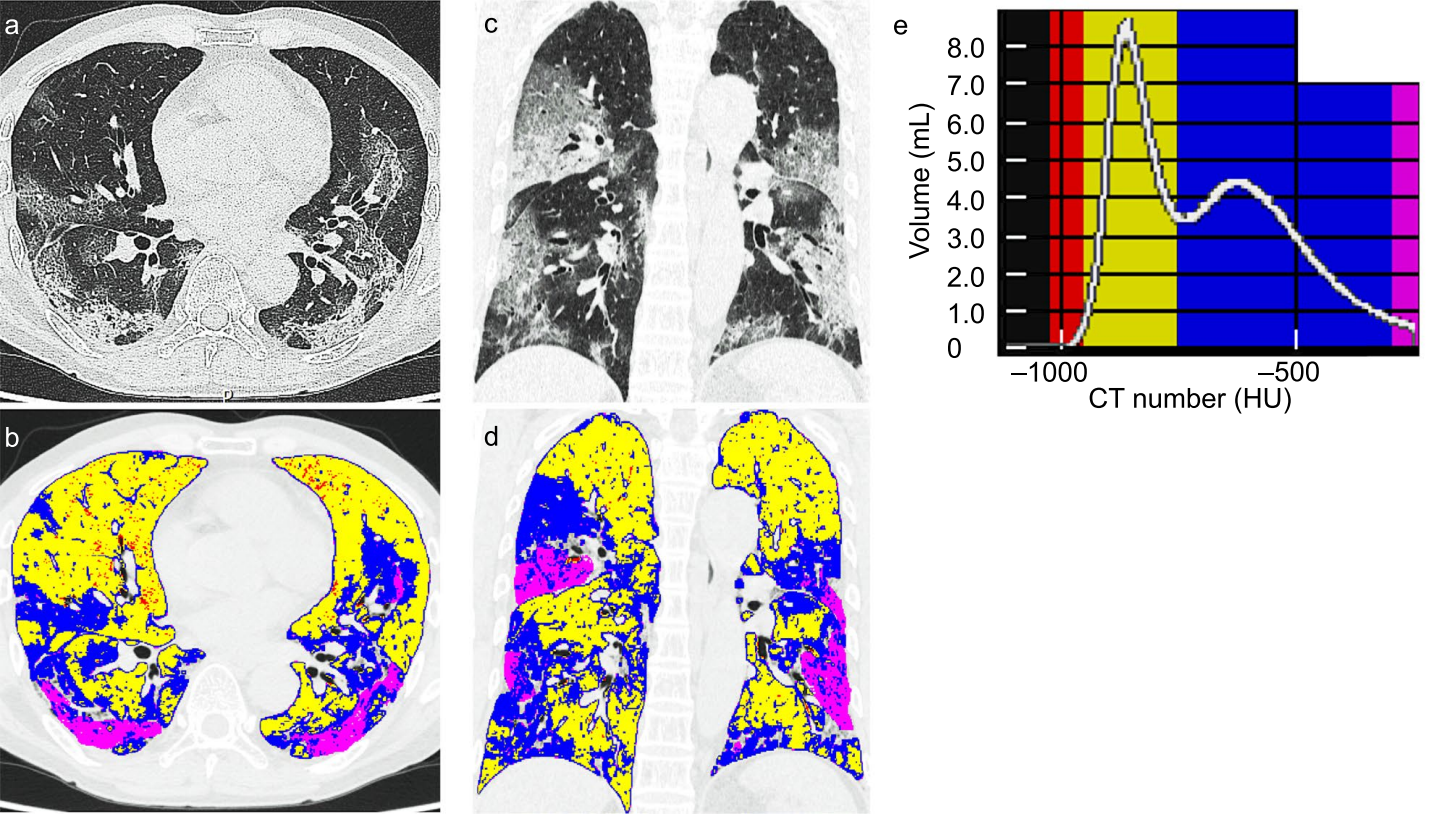
An example of the automated quantification of disease progression of COVID-19 pneumonia on CT. (**a**–**d**) CT findings of COVID-19 pneumonia by pre-setting a threshold value of Hounsfield Units and a color in order to categorize both lungs as emphysema (− 1024/− 950; red), healthy lung parenchyma (− 949/− 750; yellow), GGO (− 749/− 300; blue), or consolidation (− 299/40; violet). (**a**,**b**) Axial section. (**c**,**d**) coronal section. (**b**,**d**) Automated segmentation using an image processing tool. (**e**) Automated calculation of the volume and percentage of the impaired area, i.e. GGO and consolidation, relative to the whole lung. *COVID-19* coronavirus disease 2019, *GGO* ground-glass opacity, *HU* Hounsfield unit.

The example shown in Fig. 2 indicates how the automatic image processing tool analyzes the chest CT results and indicates the patient’s pulmonary status. The volume in each region and its percentage in the total lung are automatically calculated, enabling the quantification of the percentage of lung parenchyma damaged by COVID-19 pneumonia.

### Statistical analyses

#### Univariate analyses

Pearson’s Chi-squared test and Fisher’s exact test were used to compare each categorial variable in the severe and non-severe groups. Pearson’s Chi-square test was used if the overall number of cases was more than 40, and Fisher's exact test was used for other cases. The *t* test and Mann–Whitney's *U* test were used to compare between two unpaired groups with normality and non-normality continuous variables as outcomes, respectively. In the *t* test, Student's *t* test was used when there was equal variance in the comparison group, and Welch's *t* test was used when there was not.

#### Multiple regression analyses

We used a multiple regression analysis to quantitatively assess the clinical factors associated with the extent of GGO and consolidation within total lung by COVID-19 pneumonia observed on chest CT at admission. The proportion of GGO and consolidation extent within total lung was quantified by image analyses. We set the percentage of total lung of GGO and consolidation as the objective variables and factors associated with the severity of COVID19 pneumonia as the explanatory variables.

The number of variables included in a multivariate analysis is known to depend on the number of cases^36^. Since the number of explanatory variables that can be analyzed in a multiple regression analysis is generally considered to be the total number of cases divided by 15, the upper limit was estimated to be around 30 (450 divided by 15). The variables to be assessed in the multivariate analysis were determined before the analysis was performed. As variables associated with the severity of COVID-19 pneumonia, we selected 15 variables reported in a previous study to be associated with the progression of COVID-19 pneumonia to ARDS^2^. These included 3 categorical variables (i.e. sex, comorbidity [diabetes mellitus, hypertension]) and 12 continuous variables (i.e. age, body mass index [BMI], lactose dehydrogenase [LDH], C-reactive protein [CRP], ferritin, D-dimer, albumin, aspartate aminotransferase [AST], alanine aminotransferase [ALT], white blood cell counts, neutrophil counts, lymphocyte counts). The duration from COVID-19 infection to admission was also added as an explanatory variable because it has been reported that the shade of COVID-19 pneumonia on chest CT changes based on the time since the onset of the disease^37^. When conducting the multiple regression analysis, the following points were kept in mind: the proportion of GGO and consolidation for the total lung volume and continuous variables were log-transformed to approximate a Gaussian distribution. The multicollinearity between each variable was assessed by the variance inflation factor (VIF) < 10. The normality of the residuals of the liner regression model was confirmed by residuals versus fitted plot and a normal quantile–quantile (Q–Q) plot. Since the partial regression coefficient (B) and standardized partial regression coefficient (β) for each continuous variable obtained from a multiple regression analysis are log-transformed values, the logarithm was removed by transforming B and β by a power of 10, and these numbers were presented again as the B and β.

#### Propensity score matching

Propensity score matching was performed to adjust the background factors between the groups with non-severe and severe COVID-19 pneumonia. The background factors to be adjusted were selected from those that have been previously reported to be associated with the severity of COVID-19 infection. Because the explanatory variables that we included in the multiple regression analysis described above are associated with the development of ARDS due to COVID-19 infection, variables that did not show significant differences in the analysis were also included in the propensity score matching as background factors for adjusting. In addition to these variables, we also included creatinine, which has been reported as a factor contributing to the severity of COVID-19 in large cohort studies^6^. Because the usage of systemic corticosteroid and antivirals have been associated with COVID-19 severity^2^, medications administered at admission (i.e. systemic corticosteroid, remdesivir, tocilizumab, and baricitinib) were also included as background factors for adjusting.

We used the logistic regression analysis to evaluate the propensity score. The caliper value for this study was set to a value of 0.2 times the standard deviation of the propensity score^38^. The matching ratio was set at one-to-one matching, which is known to have the least loss of power even with a significant decrease in the number of cases. Since the *P*-value with *t* tests or Pearson’s Chi-squared test was not recommended to be used to assess the balance between groups after propensity score matching, a standardized difference < 0.1 was used for this evaluation^39^.

#### Multivariate Cox proportional hazard analyses

A multivariate Cox proportional hazards analysis was performed to estimate the variables associated with severe disease of patients infected COVID-19 in the matched cohort. Multivariate Cox proportional hazards analyses can analyze the covariates of the number of cases with an outcome divided by 10^40^. In this study, that number in the Cox proportional hazards analysis was 51 (i.e. the number of severe cases after propensity score matching) divided by 10 (result: 5). We included the 5 variables with *P* < 0.05 in the multiple regression model in the Cox proportional regression model.

Statistical analyses were conducted using Easy R (EZR) (Saitama Medical Center, Jichi Medical University, Saitama, Japan), which is a graphical user interface for R (The R Foundation for Statistical Computing, Vienna, Austria). EZR is a modified version of R commander with added biostatistical functions^41^.

## Supplementary Information

Supplementary Figure 1.

## Data Availability

The datasets used and/or analyzed during the current study available from the corresponding author on reasonable request. All data generated or analyzed during this study are included in this published article and its supplementary information files.

## Abbreviations

ALT: Alanine aminotransferase
AST: Aspartate aminotransferase
B: Partial regression coefficient
BMI: Body mass index
COPD: Chronic obstructive pulmonary disease
COVID-19: Coronavirus disease 2019
CRP: C-reactive protein
CT: Computed tomography
HR: Hazard ratio
LDH: Lactate dehydrogenase
Std: Standardized difference
VIF: Variance inflation factor
β: Standardized partial regression coefficient
95% CI: 95% Confidence interval
