# Supplementary material for: Increased lactate dehydrogenase reflects the progression of COVID-19 pneumonia on chest computed tomography and predicts subsequent severe disease
Source: Sci Rep. 2023 Jan 18;13:1012. doi: 10.1038/s41598-023-28201-2 (PMC9848045; doi:10.1038/s41598-023-28201-2)
Supplement: Supplementary file 1 — Supplementary Figure 1. [file 41598_2023_28201_MOESM1_ESM.pdf]

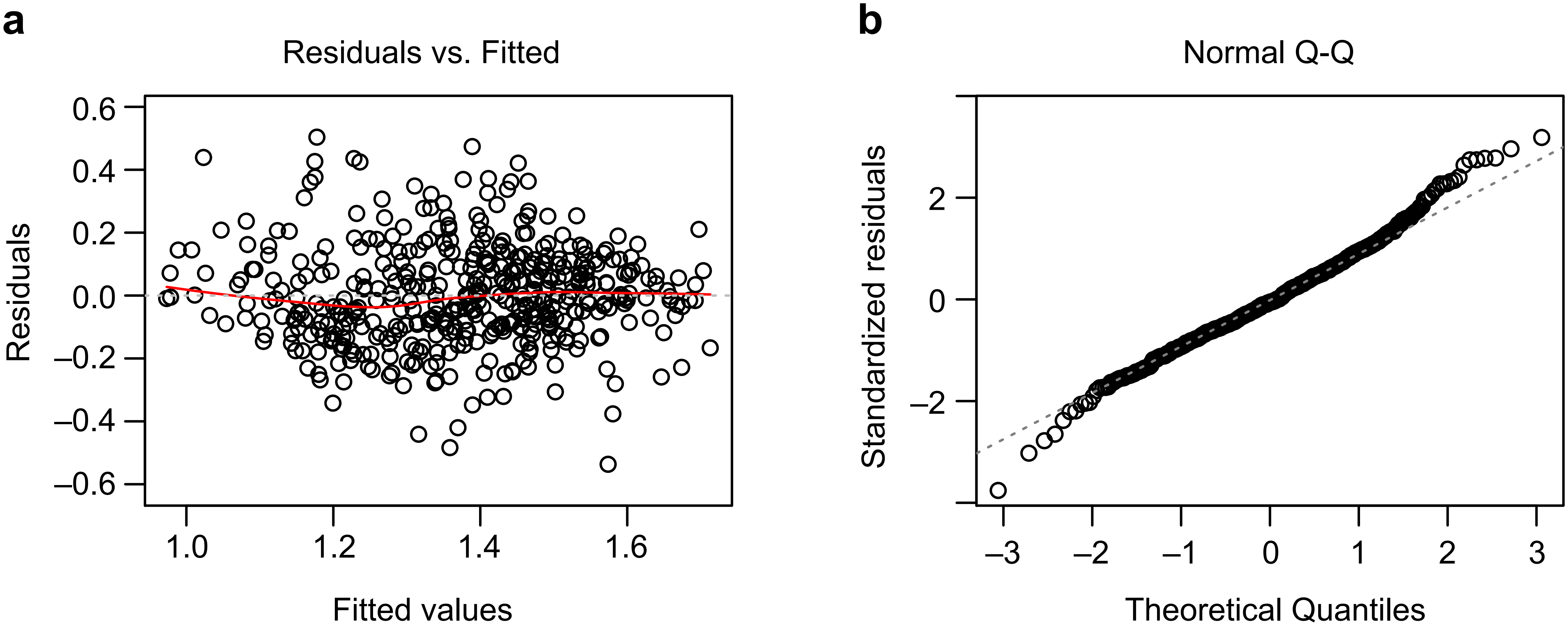

Supplementary Figure 1. Evaluation of the normality of the residuals.
The normality of the residuals was assessed with a residuals vs. fitted (a) and a normal Q-Q plot (b).
